# Radix Puerariae and Fructus Crataegi mixture inhibits renal injury in type 2 diabetes via decreasing of AKT/PI3K

**DOI:** 10.1186/s12906-017-1945-3

**Published:** 2017-09-08

**Authors:** Zhengyue Chen, Yanyan Yuan, Xinrong Zou, Mengqi Hong, Ming Zhao, Yu Zhao, Yuanping Liu, Guofu Li, Yabin Zhu, Lin Luo, Beiyan Bao, Shizhong Bu

**Affiliations:** 1 Division of Nephrology, Ningbo Urology and Nephrology Hospital, 998 Qianhe North Road, Ningbo City, 315192 Zhejiang People’s Republic of China; 2 Runliang Diabetes Laboratory, Diabetes Research Center, School of Medicine, Ningbo University, 818 Fenghua Road, Ningbo City, 315211 Zhejiang People’s Republic of China; 3 School of Medicine, Ningbo University, 818 Fenghua Road, Ningbo City, 315211 Zhejiang, People’s Republic of China

**Keywords:** Type 2 diabetes, Radix Puerariae, Fructus Crataegi, Renal injury, PI3K/AKT

## Abstract

**Background:**

Radix puerariae (RP) is a herbal medicines for diabetes, mainly because of anti-oxidative, insulin resistance and hypoglycemic effect. Fructus crataegi (FC) also possesses strong antioxidant activity in vitro. This study focused on the effects of herbal mixture of RP and FC (RPFC) on renal protection through a diabetic rat model.

**Methods:**

Type 2 Diabetic model was established with high fat diet followed by injecting rats a low dose of STZ (25 mg/kg body weight). Rats were randomly divided into five groups: normal, high fat diet, diabetes mellitus, high fat diet plus RPFC prevention, and RPFC prevention before diabetes mellitus. RPFC was given to rats daily by intragastric gavage. The blood bio-chemical index and renal pathological changes were examined. The later includes hematoxylin and eosin staining, periodic acid schiff staining, and Masson trichrome staining. Protein levels of were determined by Western blot and immunohistochemical staining. mRNA levels were detected by RT-PCR.

**Results:**

Rats prevented with RPFC resulted in decreasing blood glucose with corresponding vehicle treated rats. Glomerulus mesangial matrix expansion, renal capsule constriction, and renal tubular epithelial cell edema were less severe following RPFC prevention. Moreover, RPFC prevention reduced protein levels of PI3K, AKT, α-SMA and collagen IV in the kidney of diabetic rats.

**Conclusion:**

Combined prevention with RPFC may inhibit the PI3K/AKT pathway in the kidney, thereby prevent renal injury in diabetic rats.

## Background

Diabetic nephropathy (DN) is one of the most common microvascular complications of diabetes mellitus and is a major cause of end stage renal disease (ESRD) [1]. DN is defined as damages to the kidney caused by diabetes and mainly presents as persistent proteinuria in diabetic patients. The pathological changes mainly include glomerular mesangial expansion, basement membrane thickening, and in severe cases, glomerular sclerosis [2]. However, current therapy for DN is still limited.

Although the pathogenesis of DN is not well understood, previous studies suggest that disturbed lipid metabolism [3], abnormal hemodynamics [4], and over-produced inflammatory mediators and cytokines [5] are the major causes of DN. Moreover, a high glucose environment and changes in cytokine production can cause functional changes in mesangial cells. Increasing evidence also suggests that inflammation and fibrosis play critical roles in the development and progression of DN [6].

Traditional Chinese medicine (TCM) has been used for treating DN in China [7]. However, the exact mechanisms by which TCM relieves DN symptoms remain unknown. Radix puerariae (RP), known as ‘Gegen’ in Chinese, is the root of *Pueraria lobata* (Willd.) Ohwi or *Pueraria thomsonii* Benth (http://www.theplantlist.org), and it was shown recently to be beneficial to diabetic patients [8]. Puerarin, one of the active components of RP, has various pharmacological functions, such as eliminating oxygen free radicals, protecting myocardial cells, lowering blood pressure and inhibiting platelet aggregation [9]. It is known that puerarin can improve insulin resistance of in 3 T3-L1 lipocyte induced by free fatty acids [10]. Fructus crataegi (FC) is the fruit from *Crataegus pinnatifida* Bunge Rosaceae or *Crataegus pinnatifida var. major* N.E.Br., plants widely grown in China (http://www.theplantlist.org/). It often used to make tea in China, and the extract possesses strong antioxidant activity in vitro [11].

The exact renoprotection mechanisms of RPFC in STZ-induced diabetic rats remain unclear. We examined here whether RPFC could prevent renal injury in a rat model of DN, where progressive glomerular sclerosis and renal fibrosis was induced by a high-fat diet and a low-dose STZ.

## Methods

### Drugs and reagents

RP (the major component was purarine, then daidzin and daidzein; genistin and genistein were the least abundant) [12] and FC (essential components are flavonoids and organic acidic compounds) were provided by Shanghai Second Military Medical University. They were extracted separately using the following process: two times reflux extraction of one kilogram of each medicinal herb with 60% alcohol, the amounts of solvent of 5 folds, successively, and the extraction time of 90 min each [7]. The extract was reduced-pressure evaporated till the volume was 590 ml. RP and FC solutions were mixed at a volume ratio of 1:1 and used as the RPFC solution in this study.

### Animals and diabetic model

Male 8-week-old Sprague-Dawley (SD) rats, weighing between 250 and 300 g, were purchased from the Laboratory Animal Center of the Academy of Zhejiang Medical Sciences (Zhejiang, Certificate No. 0012371). All rats were housed at a SPF-grade laboratory animal room in the Animal Laboratory Center of Ningbo University with 20–25 °C room temperature, 50–60% humidity, a 12/12 h light/dark cycle. The Standard diet was provided by Animal Laboratory Center of Ningbo University and the high fat diet was purchased from Pu Luteng Bio-Technique Co. Ltd. The high fat diet contained 16.9% fat and 10.2% casein in the standard diet.

Rats were fed with the high fat diet (30 g per day for each rat) for 8 weeks to induce insulin resistance. The experimental diabetic model was produced by intraperitoneal injection of STZ (diluted to 1% in a 10 mmol/L citrate buffer at pH 4.5) at 25 mg/kg body weight after the rats were fasted for 12 h [13]. Rats in the control group were injected with an equal volume of the citrate buffer. Blood glucose was measured 72 h after STZ injection, and rats with blood glucose over 16.7 mmol/L were considered to be diabetic [14]. Rats were continued on the high fat diet except the rats of control group, in order to keep their blood glucose levels high.

### Experimental design

**Fig. 1:**
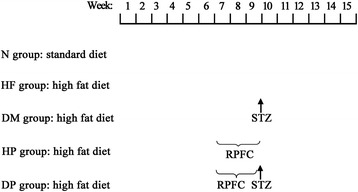
Schematic of group design during 15 weeks

The experimental procedure lasted 15 weeks, during which rats were kept on the standard or high fat diet according to their group assignments. RPFC were administered by intragastric gavage during the 7th–9th weeks, and rats not receiving RPFC were given normal saline. The day after the last administration of RPFC, STZ was injected, and rats not receiving STZ were injected with the vehicle (citrate buffer). 20 rats were randomly divided into five groups with four rats in each group: 1) Normal group, fed with the standard diet. 2) High fat diet (HF) group, fed with the high fat diet. 3) Diabetes mellitus (DM) group, fed with the high fat diet, gavaged with normal saline, and injected with STZ. 4) High fat diet plus RPFC prevention group (HP), fed with the high fat diet, gavaged with RPFC, and injected with the citrate buffer. 5) Diabetes mellitus plus RPFC prevention group (DP), fed with the high fat diet, gavaged with RPFC, and injected with STZ (Fig. 1). As increased time of high fat diet feeding, obesity and resistance to insulin would be more apparent [15]. The beta-islet cells susceptibility of STZ increase in rats feed with the high fat diet. So we add HF group to observe whether high-fat rats have metabolism disorders or renal injury, and add HP group to confirm whether RPFC has impacts on the above changes.

### Measurement of body weight, blood glucose, and urinary protein

Blood glucose was measured once a week and body weight was measured once a month. Blood samples were collected from the tail vein to measure the blood glucose level. The fasting blood glucose (FBG) was measured after a 12 h fasting, and postprandial blood glucose (PBG) was measured at 2 h after a meal. Blood glucose level was measured with One Touch Ultra test strips and blood glucose meter (Johnson& Johnson Medical Ltd., Shanghai, China).

Clinically, microalbuminuria was defined as urine albumin excretion rate (UAER) in the range of 20–200 μg/min or urine albumin at 30–300 mg/g creatinine [16]. Rats were considered to have nephropathy if they displayed microalbuminuria. Rats were housed individually in metabolic cages for 24 h to collect urinary samples analyzed with a MODULAR P800 Automation Biochemist Analyzer (Roche, Basel, SWIT).

### Oral glucose tolerance test (OGTT), and measurement of insulin and other biochemical parameters

The oral glucose tolerance test was performed on overnight-fasted rats at the end of the study. Rats were fasted for 12 h and then given a glucose solution (2 g/kg body weight), and blood samples were collected before and at 30, 60, and 120 min after the glucose solution was given [17]. Insulin and other biochemical parameters were measured at the end of study. Blood samples were collected from the femoral artery into EDTA-anticoagulant tubes, collected the serum for the detection of serum insulin, total protein (TP), high-density lipoprotein cholesterol (HDL-C), low-density lipoproteins (LDL-C), total cholesterol (TC), triglycerides (TG), ureanitrogen (BUN), creatinine (CREA) and uric acid (UA). The levels of serum insulin were measured with a Rat Insulin ELISA kit (Qiaodu, Shanghai, China). Other biochemical indicators were measured with a MODULAR P800 Automation Biochemist Analyzer (Roche, Basel, SWIT). At last, rats were sacrificed and kidney tissues were saved standby.

### Histological examination of the kidney

Renal tissues were fixed for 48 h, dehydrated through a graded series of ethanol, embedded in paraffin wax, and cut into 3 μm sections. The sections were subjected to hematoxylin and eosin (H&E), periodic acid schiff (PAS), and Masson trichrome staining and observed under a microscope.

### Real time reverse transcriptase-polymerase chain reaction (RT-qPCR)

Total RNA was extracted from renal tissues using TRIzol reagent (Invitrogen, USA). 1 microgram of total RNA was for reverse transcription. Polymerase chain reactions were performed in a Lightcycler 480 II Authorized Thermal Cycler (Roche, Basel, SWIT) using the following protocol: denaturation at 95 °C for 5 min, followed by 45 cycles of 95 °C 10 s, annealing 20 s, and 72 °C 30 s [18]. The primers used were as follows: α-SMA, F: 5′-CATTGCTGACAGGATGCAGAA-3′, and R: 5′-GAAGCATTTGCGGTGGACAA-3′; collagen IV, F: 5′-GTTGGTCTACCGGGACTCAA-3′, and R: 5′-GTTGGTCTACCGGGACTCAA-3′; β-actin, F: 5′-CTGAACCCTAAGGCCAACCG-3′, and R: 5′-GACCAGAGGCATACAGGGACAA-3′. Relative mRNA levels were determined with the 2 − ^△△^Ct method using the gene β-actin as the internal reference.

### Western blot analysis

Renal tissues of 40 mg were lysed for protein extraction. Samples (50 μg) were separated by 8% sodium dodecyl sulfate-polyacrylamide gel electrophoresis (SDS-PAGE) gels and transferred to polyvinylidene fluoride membranes. The membranes after blocked were incubated with primary antibodies to Phosphoinositide 3-kinase (PI3K) (p85) (Abcam, Cambridge, MA, 1:1000), protein kinase B (AKT) (Signalway Antibody LLC, Maryland, USA, 1:1000), and α-SMA (Abcam, Cambridge, MA, 1:200) at 4 °C overnight. Then incubated with a HRP-labeled secondary antibody (Beyotime, China). The immunoreactions were detected with a gel imaging and analysis system (Tanon, Shanghai, China). The density values of bands were quantified using the software Image J (NIH, Maryland, USA).

### Immunohistochemical staining

Renal slides (3 μm) were used to perform immunohistochemical staining of α-SMA and collagen IV on renal tissue sections [19] with the following primary antibodies: monoclonal mouse anti-rat α-SMA (Abcam, Cambridge, MA, 1:50) and polyclonal rabbit anti-rat collagen IV (Abcam, Cambridge, MA, 1:200). Quantitative analysis of the brown positive staining in glomerulus and tubules was performed using Image-Pro Plus 6.0 (Media Cybernetics, USA).

### Statistical analysis

All the data are presented as mean ± standard error of the mean (S.E.M.). The differences among groups were analyzed by randomized block design analysis of variance by using SPSS (version 13.0) software. *P* values below 0.05 were considered statistically significant.

## Results

### Blood glucose, body weight, and urinary protein measurement and OGTT

**Fig. 2:**
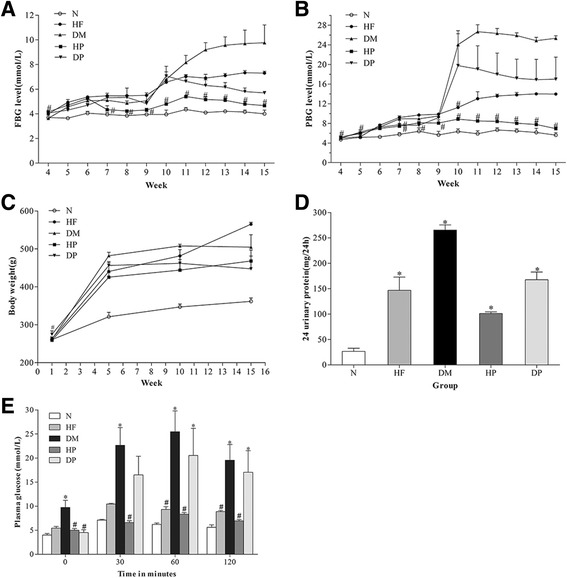
**a**, FBG levels. **b**, PBG levels from 4th week to 15th week. **c**, Body weight in each month. **d**, 24 h urinary protein at the end of the study. **e**, the result of OGTT of all rats. Results are presented as mean ± SEM, *n* ≥ 3. ^#^ indicates *p* > 0.05 compared with the normal group in Fig. 2a, b & e. * indicates *p* < 0.05 compared with the normal group in Fig. 2d & e

All the rats were alive at the end of study. FBG levels were measured from the 4th week till the end of the study (Fig. 2a). The rats in HF, DM, and DP groups showed higher FBG levels from the 5th to the 15th week than the normal group. After the injection of STZ, both FBG and PBG levels in both the DM and DP groups rose rapidly. However, FBG levels in the DP group declined slowly, and were even lower than in HF group from the 10th week to the 15th week. However, FBG levels as well as PBG levels in the HP group started to decrease from the 7th week and became lower than those in the HF group and comparable to those in the normal group. PBG levels 2 h after a meal were also measured starting from the 4th week (Fig. 2b). The rats in the HF, DM and DP groups showed higher PBG levels from the 8th to the 15th week than the normal group. However, from the 10th week, PBG levels in the DP group declined slowly, and were significantly higher than those in the HF group but lower than those in the DM group till the end of the study. This could be due to RPFC prevented effectively the increase in serum glucose in the model.

As shown in Fig. 2c, body weights increased significantly in the HF, DM, DP and HP groups as compared with the normal group. Compared with the normal group, urinary protein was significantly higher in all other groups. Moreover, urinary protein in the DP group was lower than in the DM group; similarly, urinary protein in the HP group was lower than in the HF group (Fig. 2d).

Figure 2e shows that in the OGTT, blood glucose in the normal and HF groups reached peak levels at 30 min after the rats were given oral glucose, while the other three groups reached peak levels at 60 min. Rats in the DM and DP groups showed significantly lower degrees of glucose intolerance compared with rats in the normal group.

### Biochemical parameters

Table 1 shows the measurement results of biochemical parameters in all rats. Compared with the normal group, LDL-C and TC levels were significantly different in the DM group (*p* < 0.05), and TC level was significantly increased in the other four groups, and HDL-C level was significantly increased in the HF and DP groups. These data indicate that oral administration of RPFC may improve lipid metabolism.

**Table 1:** Biochemical parameters for all rats at the end of the study

	N	HF	DM	HP	DP
TP	66.60±4.24	80.77±7.45	64.47±7.06	74.50±1.92	78.17±6.09
HDL-C	0.86±0.20	1.39±0.31*	1.13±0.11	1.05±0.14	1.36±0.28*
LDL-C	0.56±0.02	0.59±0.04	0.71±0.07*	0.62±0.04	0.58±0.04
TC	1.62±0.01	2.29±0.33*	2.81±0.04*	1.91±0.25*	2.55±0.03*
TG	2.43±1.73	2.77±0.68	2.47±0.21	2.75±0.32	2.53±1.08
BUN	5.40±0.86	5.73±0.51	5.25±0.78	5.66±0.28	5.95±0.64
CREA	23.00±4.24	21.00±2.65	23.00±2.00	20.00±1.00	21.50±0.71
UA	76.50±17.68	129.33±37.31	103±14.14	127.67±47.26	97.50±13.44

*Indicates *p* < 0.05 between other groups versus the normal group

### Renal histology

**Fig. 3:**
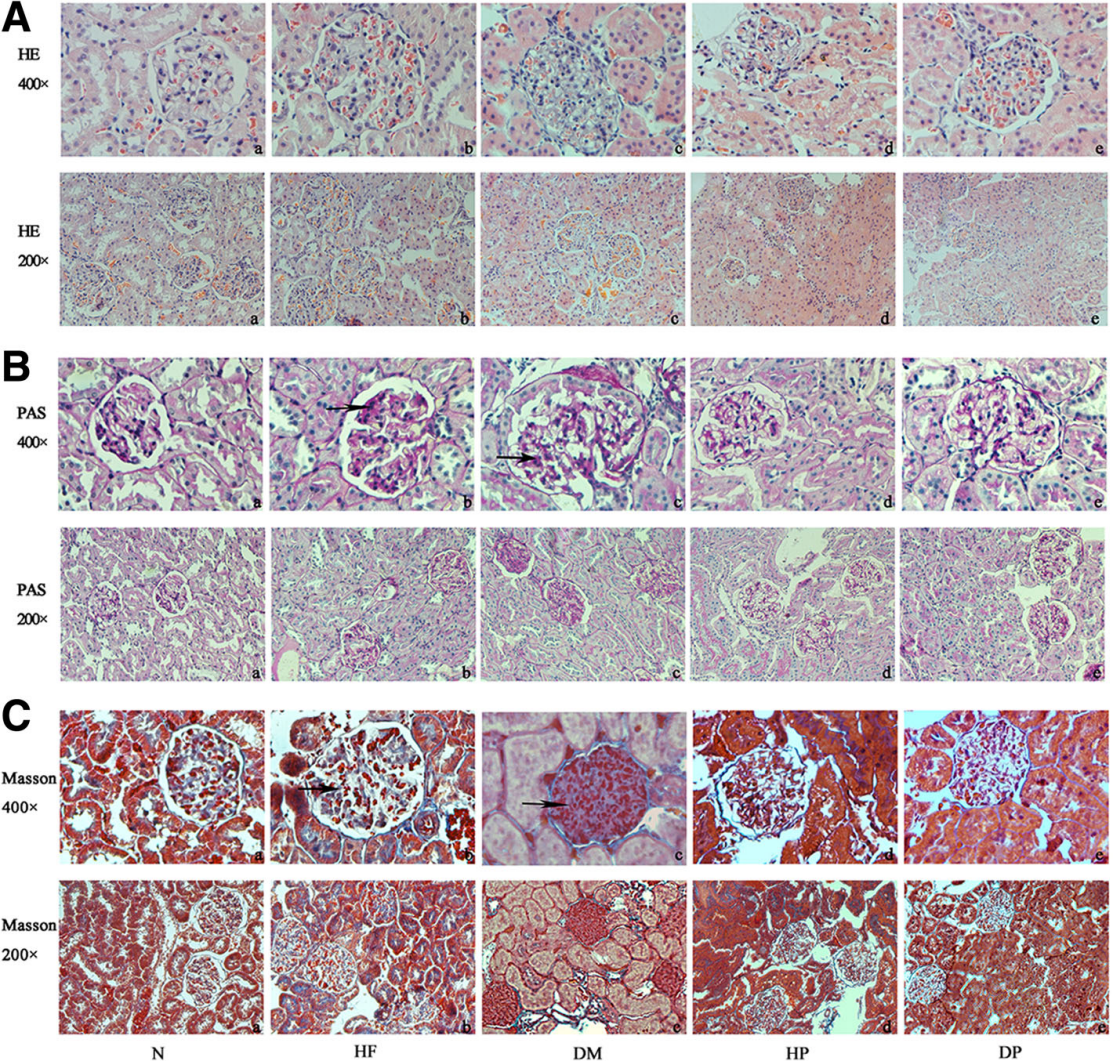
Renal pathology of experimental rats. Representative pictures of H + E, PAS, and masson trichrome staining are presented as **a** to **c**, respectively. Picture **a** represents the normal group, picture **b** represents the HF group, picture **c** represents the DM group, picture d represents the DP group, picture e represents the HP group

Glomerular hypertrophy was observed in the HF group, as glomerular extracellular matrix accumulation and renal capsule constriction, indicators of early DN, were evident in the DM group. So, renal injury was more severe in the DM group than those in the HF group. Mesangial matrix expansion and renal capsule constriction were visible in HP and DP groups, but were much less severe than in the corresponding RPFC untreated groups (Fig. 3a).

The hyperplastic state of collagen was observed by masson trichrome staining and glycogen was observed by PAS staining (Fig. 3b and c). The sedimentary hepatin granules (arrow) were observed in HF and DM groups (Fig. 3b), and collagen deposition (arrow) was particularly evident in DM groups (Fig. 3c). In addition, the renal tubular appeared foamy in the HF and DM groups because of the accumulation of fats and glycogen, while RPFC treatment reduced the accumulation of fat and glycogen in the kidney.

### Expression of PI3K and AKT in renal tissues in rats

**Fig. 4:**
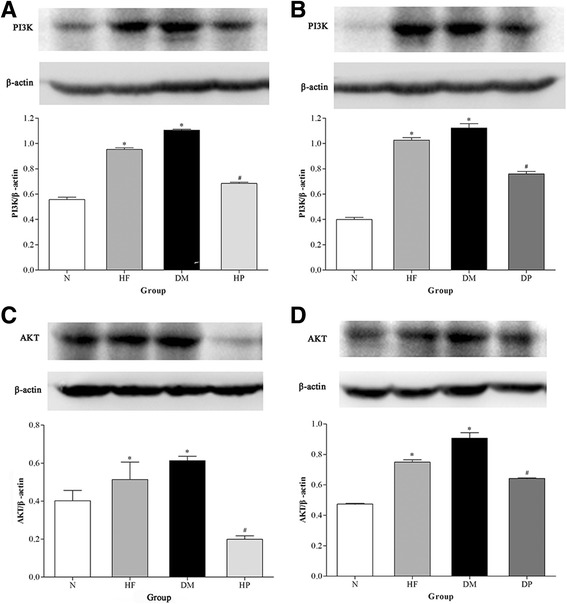
Supplement of RPFC decreased PI3K and AKT in high fat fed rats and DM rats. Protein expression of PI3K **a** and **b**, and AKT **c** and **d**. Renal PI3K and AKT increased in the kidneys of the HP and DM group rats in comparison to that of the normal group (*P < 0.05). Renal AKT decreased in the kidneys of the HP and DP group rats in comparison to that of the HF and DM group (^#^ *p* < 0.05). Bars represent mean ± SD from three experiments

PI3K and AKT play pivotal roles in regulating cell proliferation and apoptosis in the kidney [20]. We quantified the expression levels of PI3K and AKT by Western blot analysis. As shown in Fig. 4, the protein levels of PI3K and AKT were significantly higher (*P* < 0.05) in rats of the HF and DM groups than in the control rats. However, the protein levels of PI3K and AKT were lower in rats of the HP and DP groups than in the corresponding RPFC untreated groups (*P* < 0.05, Fig. 4). These results suggest that RPFC might protect against renal injury by decreasing the expression levels of PI3K and AKT proteins in diabetic rats.

### Expression of α-SMA and collagen IV

**Fig. 5:**
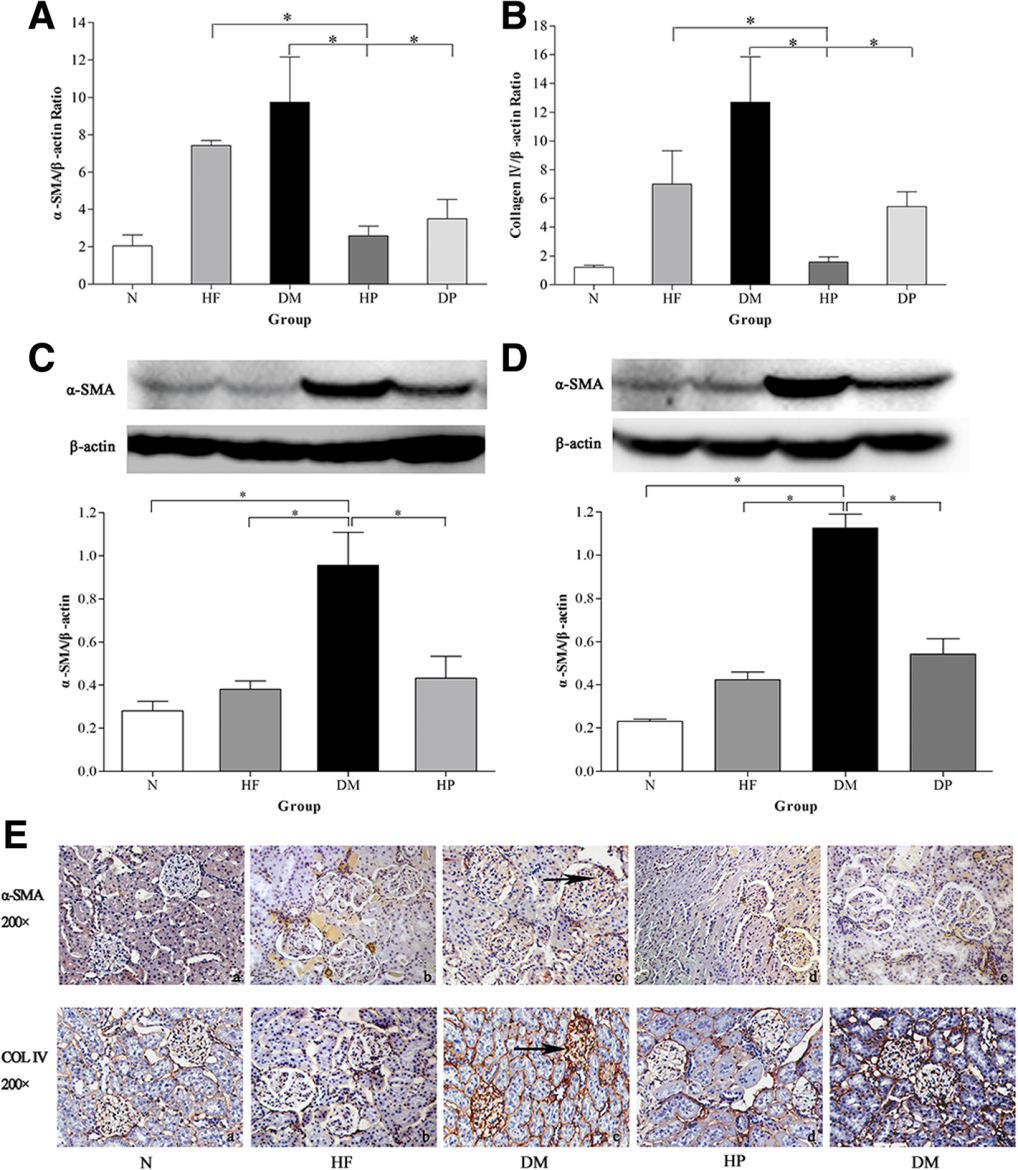
The expression of α-SMA and collagen IV in renal. The mRNA expression of α-SMA **a** and collagen IV **b**; the protein expression of α-SMA determined by Western blot **c** and **d** and immunohistochemical **e**; the protein expression of collagen IV determined by immunohistochemical **f**. The data are presented as mean ± SEM, *n* ≥ 3, *p < 0.05

As shown by real-time PCR, prevention with RPFC in rats of the DP and HP groups significantly reduced the expression of α-SMA and collagen IV as compared with the DM group. Moreover, there was a significant difference between the HF group and HP group (*P < 0.05, Fig. 5a and b). The protein levels of α-SMA was significantly higher (*P* < 0.05) in the DM group than in the normal group (Fig. 5c and d). The expressions of both α-SMA and collagen IV proteins in the DM group were increased (Fig. 5e). These data suggest that RPFC might decrease the protein levels of α-SMA and collagen IV in diabetic rats thus alleviating renal injury.

## Discussion

Human beings are heading for a health disaster with rising obesity levels and the increasing incidence of type 2 diabetes. However, some studies suggest diabetic nephropathy occupies for approximately 20 to 30% of diabetic. The incidence of diabetes and DN have seen a rapid increase in China over the last decade [9]. The diabetic nephropathy patients have a higher mortality, thus, effective preventive and adequate treatment are of uttermost importance. Despite the lifestyle interventions (such as smoking cessation, moderation of alcohol intake, healthy diet, weight control and physical activity) which can help to prevent progression or occurrence of diabetic nephropathy impairment. However, the current treatment of using ACEI and ARBs are not effective against DN [21]. In recent years, several studies have shown that TCM can ameliorate renal injury in diabetic rats [14]. In our present study, RP and FC with high specific curatorial value are both natural medicine in plants. Puerarin has exhibited classic estrogen-like biological activities and shown remarkable cardiovascular protective effects in in vivo and in vitro experiments. It improves insulin resistance and lowers blood sugar and lipid levels [22]. Moreover, puerarin also has demonstrated profound anti-oxidative effects. FC is edible and is often made into nutritious haw wine, tea, and sweetened hawthorn fruit rolls. The possible mechanism of these effects was thought to be that it can induce GLUT4 protein expression [23], inhibit oxidative stress [22]. In the present study, we find that RPFC reduces microalbuminuria effectively in early DN rats. Albuminuria is a prognostic marker of DN because the presence of proteins in the urine aggravates kidney damage [24]. To date, the research on FC has focused on its role in reducing blood lipids and cholesterol levels, lowering blood pressure, and anti-lipid peroxidation, such as treating cardiovascular diseases [25], but no study on FC and renal diseases has been conducted.

The α-SMA is involved in progressive renal changes [26] in both human and experimental rats [27]. For α-SMA is normally expressed at low levels in mesangial cells but not in podocytes. However, it becomes widely expressed when mesangial cells and podocytes are damaged and show phenotypic changes. Moreover, increased level of renal α-SMA in diabetic rats is associated with tubulointerstitial fibrosis involved in DN [28]. Glomerulus mesangial cells with higher expression of α-SMA show enhanced contractility, resulting in the changes of blood flow dynamics in glomerulus, which eventually lead to glomerular sclerosis [29]. Moreover, the presence of α-SMA will induce synthesis and secretion of extracellular matrix (ECM), which also leading to glomerular sclerosis. Collagen IV can be synthesized and secreted by glomerular mesangial cells, endothelial cells, epithelial cells and tubular epithelial cells, it plays a major role in renal fibrosis in progression of DN [30].

Our findings showed that the expression of α-SMA and collagen IV were significantly increased in diabetic rats, while decreased with prevention of RPFC. The preventive activity of RPFC in diabetic rats was partly mediated by inhibition of α-SMA expression, which was consistent with previous studies showing that the high glucose milieu of diabetes can increase the synthesis of α-SMA [27]. Moreover, it was reported that rats with diabetes exhibited markedly increased renal Smad1 and collagen IV expression and extracellular matrix deposition [31]. These data suggest that RPFC might play a role in inhibiting PI3K/AKT signaling pathway, which indirectly leads to reduction of α-SMA and collagen IV.

In our previous study, RPFC did not affect normal rats. We also found that there is no difference in serum insulin levels among the five groups (data not shown). Moreover, we found that RPFC decreased blood glucose level not only under hyperglycemic but also under hyperlipidemia conditions. A recent research [32] demonstrated that puerarin had more pronounced antioxidative effect than losartan. In addition, it was demonstrated that puerarin could attenuated proteinuria and podocyte injury in diabetic rats probably through reduction of oxidative stress in the diabetic kidney [32]. Similarly, our current study suggests that RPFC might target PI3K/AKT pathway to protect renal tissues and delay the progression of DN.

Here we provide potential mechanisms to explain how herbal mixture of RP and FC affects rats with early DN. It would also be necessary to determine whether RPFC could help ameliorate renal injury in patients with more advanced DN and with other nephropathies in future studies. Based on the current study, RPFC is safe and no side effects were observed. One traditional Chinese medicine research in mice and rats has observed acute and long-term toxicity of crude flovone of pueraria gavaged for 90 days, points out that crude flovone of pueraria has no accumulated toxicity. However, it is not known whether long-term treatment with RPFC would cause any severe side effects. It has been known that RP is not suitable for people have gastritis diseases, and RP given intravenously could cause itching and nausea, while FC is not good for anyone who has digestive diseases.

## Conclusion

In summary, our study demonstrated for the first time that RPFC was effective in preventing early DN in rats, possibly by suppressing PI3K/AKT signaling and repressing the expression of α-SMA and collagen IV in glomerulus.

## Abbreviations

FC: Fructus crataegi
RP: Radix puerariae

## References

[1] Hosseini SM, Boright AP, Sun L, Canty AJ, Bull SB, Klein BE, Klein R, Paterson AD, Group DER (2015). The association of previously reported polymorphisms for microvascular complications in a meta-analysis of diabetic retinopathy. Hum Genet.

[2] Dalla Vestra M, Saller A, Mauer M, Fioretto P (2001). Role of mesangial expansion in the pathogenesis of diabetic nephropathy. J Nephrol.

[3] Herman-Edelstein M, Scherzer P, Tobar A, Levi M, Gafter U (2014). Altered renal lipid metabolism and renal lipid accumulation in human diabetic nephropathy. J Lipid Res.

[4] Kanauchi M, Ishii K, Nishiura K, Dohi K (1994). Effect of exercise on hemodynamics and urinary protein excretion in patients with early-stage diabetic nephropathy. Nihon Jinzo Gakkai Shi.

[5] Duran-Salgado MB, Rubio-Guerra AF (2014). Diabetic nephropathy and inflammation. World J Diabetes.

[6] Ma J, Wu H, Zhao CY, Panchapakesan U, Pollock C, Chadban SJ (2014). Requirement for TLR2 in the development of albuminuria, inflammation and fibrosis in experimental diabetic nephropathy. Int J Clin Exp Pathol.

[7] Fu X, Song B, Tian GW, Li JL (2014). The effects of the water-extraction of Astragali radix and Lycopi herba on the pathway of TGF-smads-UPP in a rat model of diabetic nephropathy. Pharmacogn Mag.

[8] Hou Q, Ao X, Li G, Zhang Y (2012). Puerarin combined with avandia for diabetic nephropathy. Zhong Nan Da Xue Xue Bao Yi Xue Ban.

[9] Jin G, Yang P, Gong Y, Fan X, Tang J, Lin J (2009). effects of puerarin on expression of apelin and its receptor of 2K1C renal hypertension rats. Zhongguo Zhong Yao Za Zhi.

[10] Zhao Y, Zhou Y (2012). Puerarin improve insulin resistance of adipocyte through activating Cb1 binding protein path. Chin J Integr Med.

[11] Chen J, Zhao H, Yang Y, Liu B, Ni J, Wang W (2011). Lipid-lowering and antioxidant activities of Jiang-Zhi-Ning in traditional Chinese medicine. J Ethnopharmacol.

[12] Chen TR, Chen LA, Wei QK (2010). Evaluation of quality of radix Puerariae herbal medicine by isoflavonoids. J Pharm Pharmacol.

[13] Sugano M, Yamato H, Hayashi T, Ochiai H, Kakuchi J, Goto S, Nishijima F, Iino N, Kazama JJ, Takeuchi T (2006). High-fat diet in low-dose-streptozotocin-treated heminephrectomized rats induces all features of human type 2 diabetic nephropathy: a new rat model of diabetic nephropathy. Nutr Metab Cardiovasc Dis.

[14] Zhang H, Zhao T, Gong Y, Dong X, Zhang W, Sun S, Wang H, Gu Y, Lu X, Yan M (2014). Attenuation of diabetic nephropathy by Chaihuang-Yishen granule through anti-inflammatory mechanism in streptozotocin-induced rat model of diabetics. J Ethnopharmacol.

[15] Tancrede G, Rousseau-Migneron S, Nadeau A (1983). Long-term changes in the diabetic state induced by different doses of streptozotocin in rats. Br J Exp Pathol.

[16] Jung CH, Jung SH, Kim KJ, Kim BY, Kim CH, Kang SK, Mok JO (2014). Differential associations of central and brachial blood pressure with carotid atherosclerosis and microvascular complications in patients with type 2 diabetes. BMC Cardiovasc Disord.

[17] Xu Z, Ju J, Wang K, Gu C, Feng Y (2014). Evaluation of hypoglycemic activity of total lignans from Fructus Arctii in the spontaneously diabetic Goto-Kakizaki rats. J Ethnopharmacol.

[18] Koppelman MH, Cuypers HT, Emrich T, Zaaijer HL (2004). Quantitative real-time detection of parvovirus B19 DNA in plasma. Transfusion.

[19] Zhang W, Zhao L, Su SQ, Xu XX, Wu YG (2014). Total glucosides of paeony attenuate renal tubulointerstitial injury in STZ-induced diabetic rats: role of toll-like receptor 2. J Pharmacol Sci.

[20] Obeidat M, Li L, Ballermann BJ (2014). TIMAP promotes angiogenesis by suppressing PTEN-mediated Akt inhibition in human glomerular endothelial cells. Am J Physiol Renal Physiol.

[21] Ren F, Tang L, Cai Y, Yuan X, Huang W, Luo L, Zhou J, Zheng Y (2015). Meta-analysis: the efficacy and safety of combined treatment with ARB and ACEI on diabetic nephropathy. Ren Fail.

[22] She S, Liu W, Li T, Hong Y (2014). Effects of puerarin in STZ-induced diabetic rats by oxidative stress and the TGF-beta1/Smad2 pathway. Food Funct.

[23] Song CY, Bi HM (2004). effects of puerarin on plasma membrane GLUT4 content in skeletal muscle from insulin-resistant Sprague-Dawley rats under insulin stimulation. Zhongguo Zhong Yao Za Zhi.

[24] Jim B, Santos J, Spath F, Cijiang He J (2012). Biomarkers of diabetic nephropathy, the present and the future. Curr Diabetes Rev.

[25] Liu LT, Zheng GJ, Zhang WG, Guo G, Wu M (2014). clinical study on treatment of carotid atherosclerosis with extraction of polygoni cuspidati rhizoma et radix and crataegi fructus: a randomized controlled trial. Zhongguo Zhong Yao Za Zhi.

[26] Villanueva S, Contreras F, Tapia A, Carreno JE, Vergara C, Ewertz E, Cespedes C, Irarrazabal C, Sandoval M, Velarde V (2014). Basic fibroblast growth factor reduces functional and structural damage in chronic kidney disease. Am J Physiol Renal Physiol.

[27] Niu H, Nie L, Liu M, Chi Y, Zhang T, Li Y (2014). Benazepril affects integrin-linked kinase and smooth muscle alpha-actin expression in diabetic rat glomerulus and cultured mesangial cells. BMC Nephrol.

[28] Liu P, Li F, Qiu M, He L (2014). Expression and cellular distribution of TLR4, MyD88, and NF-kappaB in diabetic renal tubulointerstitial fibrosis, in vitro and in vivo. Diabetes Res Clin Pract.

[29] Suwanpen C, Nouanthong P, Jaruvongvanich V, Pongpirul K, Pongpirul WA, Leelahavanichkul A, Kanjanabuch T (2016). Urinary podocalyxin, the novel biomarker for detecting early renal change in obesity. J Nephrol.

[30] Zeisberg M, Ericksen MB, Hamano Y, Neilson EG, Ziyadeh F, Kalluri R (2002). Differential expression of type IV collagen isoforms in rat glomerular endothelial and mesangial cells. Biochem Biophys Res Commun.

[31] Korish AA, Abdel Gader AG, Korashy HM, Al-Drees AM, Alhaider AA, Arafah MM (2015). Camel milk attenuates the biochemical and morphological features of diabetic nephropathy: inhibition of Smad1 and collagen type IV synthesis. Chem Biol Interact.

[32] Zhong Y, Zhang X, Cai X, Wang K, Chen Y, Deng Y (2014). Puerarin attenuated early diabetic kidney injury through down-regulation of matrix metalloproteinase 9 in streptozotocin-induced diabetic rats. PLoS One.

